# Menin mediates Tat-induced neuronal apoptosis in brain frontal cortex of SIV-infected macaques and in Tat-treated cells

**DOI:** 10.18632/oncotarget.14993

**Published:** 2017-02-02

**Authors:** Jun Wang, Yu Zhang, Qiping Xu, Jinhua Qiu, Honghua Zheng, Xiang Ye, Yuhua Xue, Yongmei Yin, Zhou Zhang, Ying Liu, Yanling Hao, Qiang Wei, Wei Wang, Kazuyasu Mori, Shuji Izumo, Ryuji Kubota, Yiming Shao, Hui Qin Xing

**Affiliations:** ^1^ Fujian Provincial Key Laboratory of Neurodegenerative Disease and Aging Research, Institute of Neuroscience, Department of Pathology, Basic Medicine, Medical College, Xiamen University, Xiamen, Fujian 361102, China; ^2^ School of Pharmaceutical Sciences at Xiamen University, Xiamen, Fujian 361102, China; ^3^ The Fifth People's Hospital of Wuxi, Affiliated to Jiangnan University, Wuxi, Jiangsu 214005, China; ^4^ State Key Laboratory for Infectious Disease Prevention and Control, Collaborative Innovation Center for Diagnosis and Treatment of Infectious Diseases, National Center for AIDS/STD Control and Prevention, Chinese Center for Disease Control and Prevention, Beijing 102206, China; ^5^ Institute of Laboratory Animal Sciences of Chinese Academy of Medical Science, Beijing 100021, China; ^6^ AIDS Research Center, National Institute of Infectious Disease, Tokyo 862-1640, Japan; ^7^ Division of Molecular Pathology, Center for Chronic Viral Diseases, Graduate School of Medical and Dental Sciences, Kagoshima University, Kagoshima 890-8544, Japan

**Keywords:** menin, Tat, neuron, apoptosis, human immunodeficiency virus-associated neurocognitive disorder

## Abstract

The molecular mechanisms involved in human immunodeficiency virus (HIV)-associated neurocognitive disorder (HAND) remain poorly understood. It has been recently reported that HIV-1 Tat transactivation requires menin, suggesting that menin may be involved in HAND pathogenesis. But the role of menin is not clear. Here, we found that protein level of menin was increased in simian-human immunodeficiency chimeric virus (SHIV)_-SF162.P4_ and simian immunodeficiency virus (SIV) _sm543-3_-infected rhesus macaques compared with the controls by immunohistochemistry (IHC) and western blot. Menin mainly expressed in the frontal cortex neurons of the brain, more importantly, the number of menin-staining cells was positively correlated with cleaved-caspase-3-positive cells while it was negatively correlated with a neuron-specific nuclear protein NeuN-positive cells, suggesting that expression of menin may induce neuronal apoptosis. Further studies showed that menin level was significantly increased during Tat-induced apoptosis, while downregulation of menin by pll3.7-MEN1-shRNA attenuated the Tat-induced cleavage of caspase-3 and caspase-8 in SY5Y cells and primary neuron cultures. Together, our findings reveal a pro-apoptotic role of menin in the brains of the SIV-infected macaques and the cultured neurons, indicating that targeting menin may be potential to block the HIV-1 Tat induced neuronal damage in HAND.

## INTRODUCTION

Human immunodeficiency virus (HIV) infection frequently results in neurological damage [1, 2], such as HIV-associated neurocognitive disorder (HAND), which was originally defined as the acquired immune deficiency syndrome (AIDS)-dementia complex based on motor, cognitive, and behavioral symptoms and signs. Currently, HAND is defined as an “HIV-associated neuro-cognitive disorder,” which is a broader spectrum graded classification based on abnormal neuropsychological testing performance and the presence or absence of patient perception of functional limitations related to cognitive impairment [3].

Recent anti-retroviral therapy (ART) has successfully reduced the progression of AIDS, although HAND remains frequent. Autopsy analyses have also reported an increasing incidence of HAND [4]. The histopathological evidences of HAND in the cerebral cortex are characterized as neuronal loss [5–7] and neuronal apoptosis [8–9]. In a previous study, we observed that apoptosis played a role in HAND in a macaque-AIDS model and in human AIDS [10–11]. However, it remains unclear that how neuronal apoptosis contributes to the pathogenesis of HIV-1-associated brain damage.

HIV-1 directly infects microglia/macrophages, but does not infect neurons in the brain. Although there is neuronal damage following HIV-1 infection, the mechanisms remain poorly understood. Recent results show that the HIV-1 regulatory protein Tat is a critical factor in the trans-neuronal damages of the virus [12–14]. It not only transactivates replication and expression of viral genes, but is also secreted from the infected cells [15]. Tat induces apoptotic death in cultured rat neurons in a time-dependent manner [16], and the injection of Tat into the brain leads to activation of glial cells, influx of inflammatory cells, induction of inducible nitric oxide synthase, and neurotoxicity *in vivo* [17–19]. However, the mechanisms have not been fully illustrated.

It was recently reported that HIV-1 Tat transactivation required menin [20], which is a 610-amino acid protein encoded by the multiple endocrine neoplasia type 1 (MEN1) gene [21]. These results suggest that menin is involved in HAND pathogenesis. However, the relationship between HIV-1 Tat and menin in HAND is unclear. More specifically, it remains to be shown whether tat-induced apoptosis is menin-dependent *in vitro* and in simian-human immunodeficiency chimeric virus (SHIV)_-SF162.P4_ and simian immunodeficiency virus (SIV)_sm543-3_-infected macaques, as well as whether menin facilitates neuronal apoptosis. Therefore, we analyzed menin expression and neuronal apoptosis in the frontal cortex of SHIV_-SF162.P4_ and (SIV)_sm543-3_-infected rhesus macaques and in cultured neurons by IHC staining, western blot, and immunofluorescence. Our findings reveal a pro-apoptotic role of menin in the brains of the SIV-infected macaques and the cultured neurons, indicating that targeting menin may be potential to block the HIV-1 Tat transaction-associated neuronal damages in HAND.

## RESULTS

### Viral RNA loads of the SIV-infected rhesus macaques

Molecularly cloned SHIV_-SF162.P4_ and SIV_sm543-3_ were used. The viral RNA loads of 13 macaques in the peripheral blood at the time of autopsy are summarized in Table 1. Six macaques (*#1-6*) were infected with SHIV_-SF162.P4_ that was inserted the HIV-related genes, and three macaques (*#7-9*) were infected with SIV_sm543-3_. These two virus strains made rhesus macaques to appear similar brain pathological changes with previous SIVmac239-infected rhesus macaques [10, 22–23]. Four macaques (*#10-13)* were used as controls. All SHIV_-SF162.P4_ and SIV_sm543-3_-infected rhesus macaques showed high viral loads, especially #7 and #8, and the macaques exhibited weight loss and became morbid at the time of autopsy.

**Table 1:** Clinical data of the macaques examine in this study

AnimalNo.	Sex	Age at virusinoculation(weeks)	Age atDeath(weeks)	Durationofinfection(weeks)	Viralinoculums	Viral RNAload in plasmaat autopsy(copies/ml)	Clinical information
1	M	132	321	189	SHIV_-SF162.P4_	67,000	Body weight loss and morbid
2	M	132	321	189	SHIV_-SF162.P4_	56,000	Body weight loss and morbid
3	F	132	295	163	SHIV_-SF162.P4_	45,000	Body weight loss and morbid
4	F	132	372	240	SHIV_-SF162.P4_	25,000	Body weight loss and morbid
5	M	132	372	240	SHIV_-SF162.P4_	78,000	Body weight loss and morbid
6	F	132	321	189	SHIV_-SF162.P4_	85,000	Body weight loss and morbid
7	M	392	469	77	SIV_sm543-3_	9,42,000	Body weight loss and morbid
8	M	304	438	134	SIV_sm543-3_	7,94,000	Body weight loss and morbid
9	M	224	335	131	SIV_sm543-3_	29,900	Body weight loss and morbid
10	F		156			Control	
11	M		210			Control	
12	M		209			Control	
13	M		253			Control	

### Protein levels of Menin were increased in the frontal cortex of SIV-infected macaques

**Figure 1:**
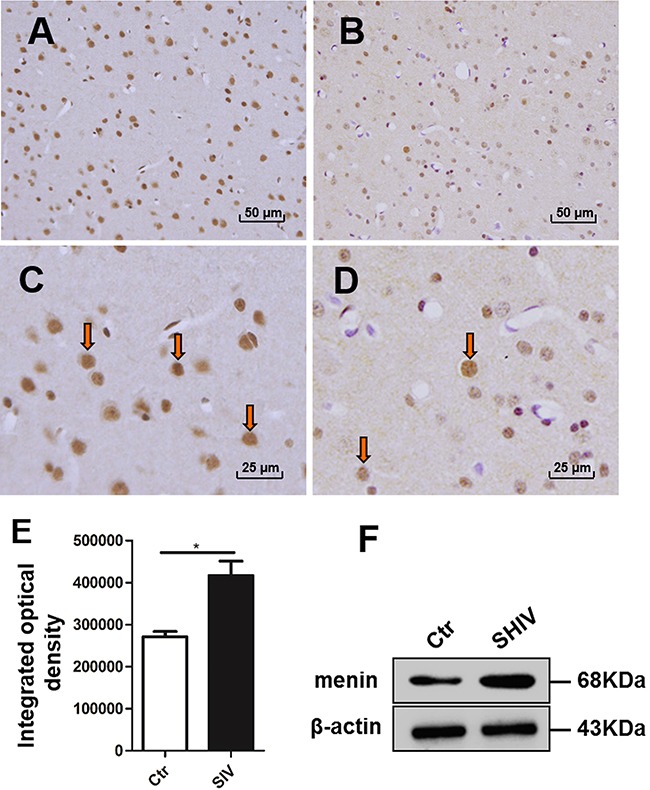
Menin were increased in the frontal cortex of SIV-infected macaques Menin expression is visible in nuclei, with some cytoplasmic expression, in the frontal cortex of SIV-infected **A**. and control **B**. macaques. There are more menin-positive cells in SIV-infected macaques **A, C**. than in the controls **B, D**. IOD analysis shows more menin immunostaining in the frontal cortex of SIV-infected macaques (*#1-#9*) compared with control macaques (*#10–13*), integrated optical density = optical density - background **E**. data are expressed as mean ± SD, *P < 0.05. Western blotting also shows increased menin expression in SHIV_-SF162.P4_-infected macaques (*#1*) compared with control macaques (*#12*). A specific β-actin band (about 43 kDa) is shown under the menin band (about 68 kDa) **F**. Original magnification: (A-B) 200×; (C, D) 400×. (A, C) from macaque *#9*, (B, D) from *#10*. Arrows showing positive cells of IHC staining.

Anti-menin immunostaining showed increased menin expression in the frontal cortex of SIV-infected macaques (Figure 1A, 1C) compared with the controls (Figure 1B, D). Menin expression was primarily observed in nuclei, with some cytoplasmic expression. Integrated optical density (IOD) analysis showed increased menin immunostaining in the frontal cortex of SHIV_-SF162.P4_ and SIV_sm543-3_-infected macaques (*#1-#9)* compared with control macaques (*#10–13*) (Figure 1E). Two randomly selected samples from a SHIV_-SF162.P4_-infected macaque (*#1*) and a control macaque (*#12*) were analyzed by western blot to detect menin expression. Western blot analysis also confirmed the IHC findings, showing significantly increased menin expression in the frontal cortex of SHIV_-SF162.P4_ -infected macaques compared with control macaques (Figure 1F).

### Menin expression was primarily observed in the nuclei of the frontal cortex neurons in SIV-infected macaques

**Figure 2:**
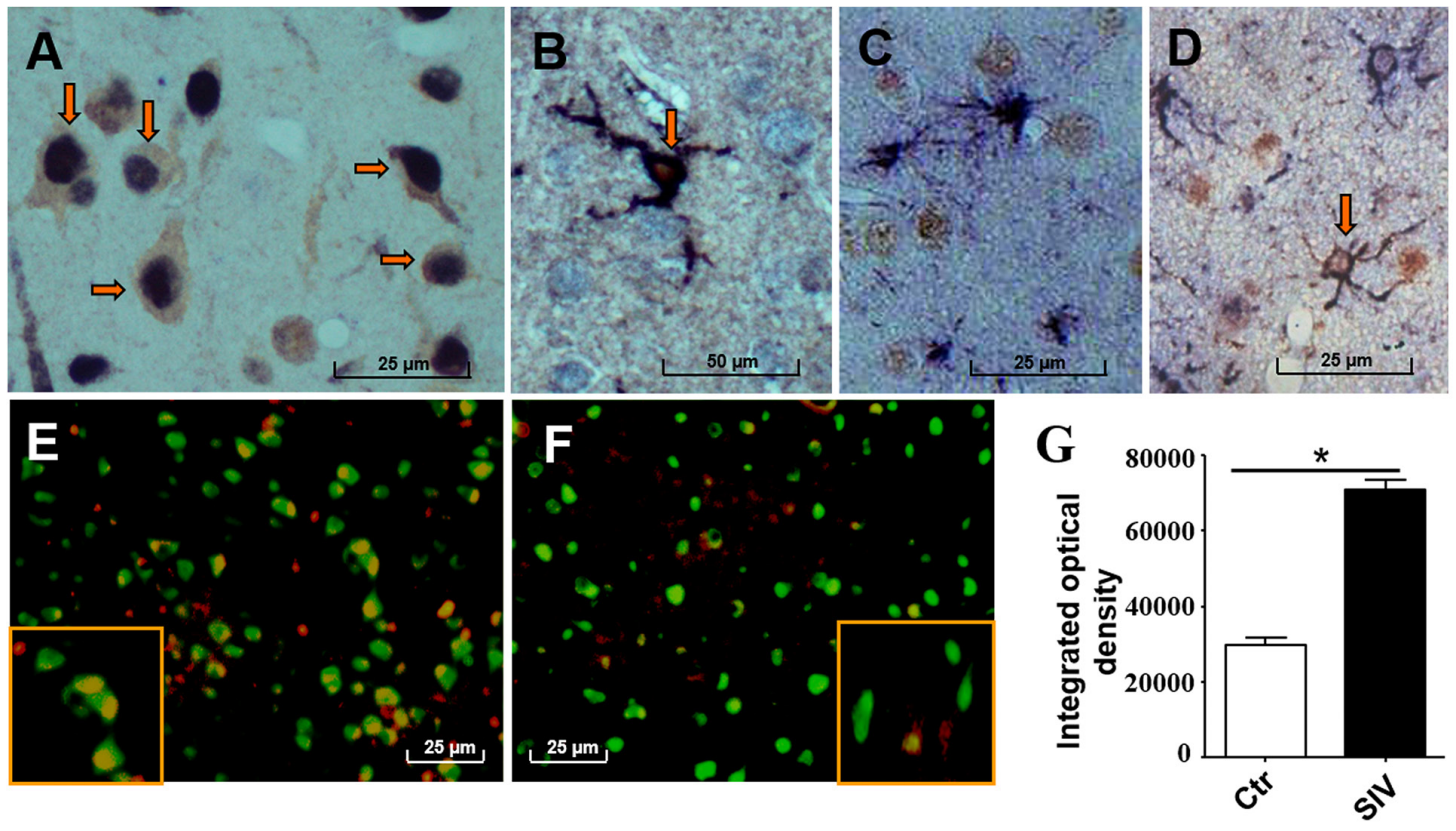
Menin expression was primarily observed in the nuclei of the frontal cortex neurons in SIV-infected macaques Menin expression (dark blue) is mainly observed in the nuclei of neurons (NeuN brown) in the frontal cortex of SIV-infected macaques **A**. Menin (dark blue) is also positive in activated microglial cytoplasm and processes, but not in microglial nuclei (Iba1, red) **B**. Menin (brown) is rarely stained in astrocytes of the cerebral cortex (GFAP, dark blue) **C**. but is positive in membranes and processes of white matter astrocytes (GFAP, dark blue) **D**. Representative double-labeled immunofluorescence images show NeuN (green) and menin (red) expression in the frontal cortex **E–F**. Menin expression is increased in SIV-infected macaques (E) compared with control macaques (F). Analysis of IOD of the double-positive area (yellow) shows significantly increased menin expression in neuronal nuclei of the SIV-infected macaques (*#2, 4, 7, 8*) compared with control macaques (*#10–13*) **G**. Data are expressed as mean ± SD, *P < 0.05. Original magnification: (A–D) 800×, (E–F) 400×. (A) From macaque *#7*; (B) from macaque *#1;* (C, D) from macaque *#3*. (E) from macaque *#7*; (F) from macaque *#13*. Arrows showing positive cells of double IHC staining.

We performed double-labeled IHC for menin and a neuron-specific nuclear protein NeuN, an astrocyte-specific cell marker GFAP, or activated microglia marker Iba1. Results showed menin expression primarily in neuronal nuclei of the frontal cortex in SIV_sm543-3_ and SHIV_-SF162.P4_-infected macaques (Figure 2A). Menin expression was also observed in activated microglial cytoplasm and spinous processes, but expression was not strong in the nuclei of these cells in SIV_sm543-3_ and SHIV_-SF162.P4_-infected macaques (Figure 2B). Menin expression was rarely observed in astrocytes in the frontal cortex (Figure 2C), but was positive in the processes of astrocytes in the white matter of the SIV_sm543-3_ and SHIV_-SF162.P4_-infected macaques (Figure 2D). Double-labeled immunofluorescence for NeuN (green) and menin (red) in the frontal cortex showed increased menin expression in SIV-infected macaques (Figure 2E) compared with control macaques (Figure 2F). IOD analysis showed significantly increased menin expression in neurons of SHIV_-SF162.P4_ and SIV_sm543-3_ -infected macaques (*#2, 4, 7, 8*) compared with control macaques (*#10–13*) (Figure 2G, *P* < 0.05).

### Menin expression was correlated with neuronal damage in SIV-infected macaques

**Figure 3:**
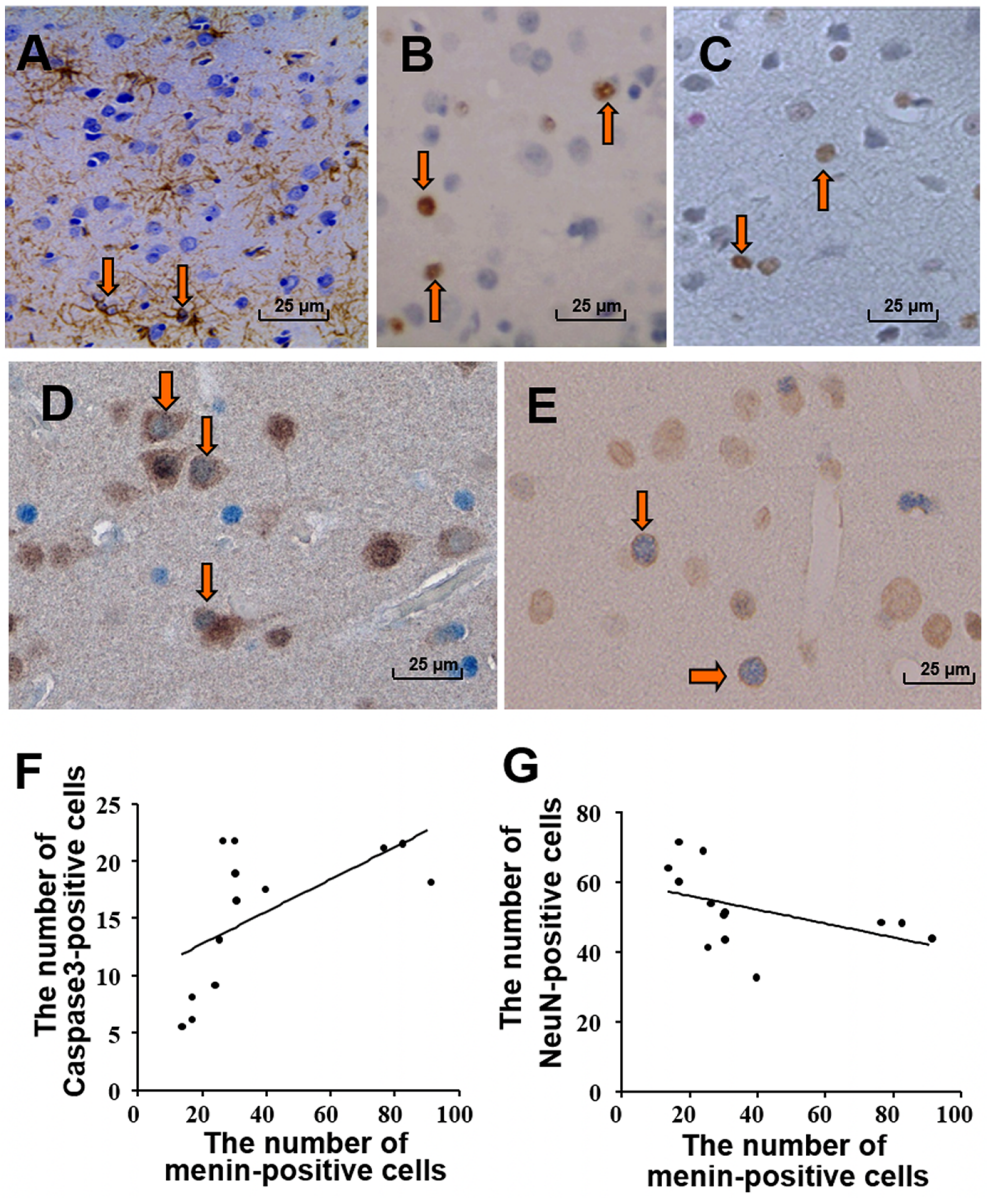
Menin expression was correlated with neuronal damage in SIV-infected macaques Astrocytic gliosis is shown in the frontal cortex with GFAP IHC **A**. TUNEL-positive cells **B**. and ssDNA-positive cells **C**. are mainly small neuronal and glial cells. Arrows showing positive cells of IHC staining (A, B, C). Apoptosis of pyramidal neurons and small neurons of the cortex is seen by double-labeled IHC for ssDNA (blue) and NeuN (brown, **D**). Menin- (brown) and cleaved-caspase 3- (dark blue) double-labeled cells **E**. Arrows showing positive cells of double IHC staining **D, E**. Significantly positive correlation (*P* = 0.0118, R = 0.6722) is demonstrated between the number of cleaved-caspase 3-positive cells and menin-positive cells in 13 macaques **F**. There is a significant negative correlation between the number of NeuN-positive cells and menin-positive cells in 13 macaques (*P* = 0.0069, R = −0.707) **G**. Original magnification: (A–E) 400×. (A) From macaque *#9*; (B-C) from macaque *#6*; (D) from *#5*; (E) from *#4*.

In our previous research, we reported glial and neuronal apoptosis in the frontal cortex of SIV-infected macaques using TUNEL and ssDNA immunostaining assays [10]. In the present study, we used different viral strains that have similar pathological features compared with previous SIVmac239 [10, 22–23]. In the SHIV_-SF162.P4_ and SIV_sm543-3_- infected macaques, gliosis was observed in cortical layers II–V using IHC against GFAP (Figure 3A). TUNEL-positive (Figure 3B) and ssDNA-positive cells (Figure 3C) were mainly observed in cortical layer II. Double-labeled IHC for ssDNA and NeuN revealed double labeling in some pyramidal neurons (Figure 3D). Double-labeled menin and cleaved-caspase 3 IHC revealed some double-positive cells in layers II–V (Figure 3E). Analysis of the relationship between the number of cleaved-caspase 3-positive cells and menin-positive cells in 13 macaques revealed a significantly positive correlation (*P* = 0.0118, R = 0.6722, Figure 3F). However, there was a significantly negative correlation between the number of NeuN-positive cells and menin-positive cells in 13 macaques (*P* = 0.0069, R = −0.7070, Figure 3G).

### Tat effectively enhances menin expression and induces apptopsis in primary neurons and SH-SY5Y cells

**Figure 4:**
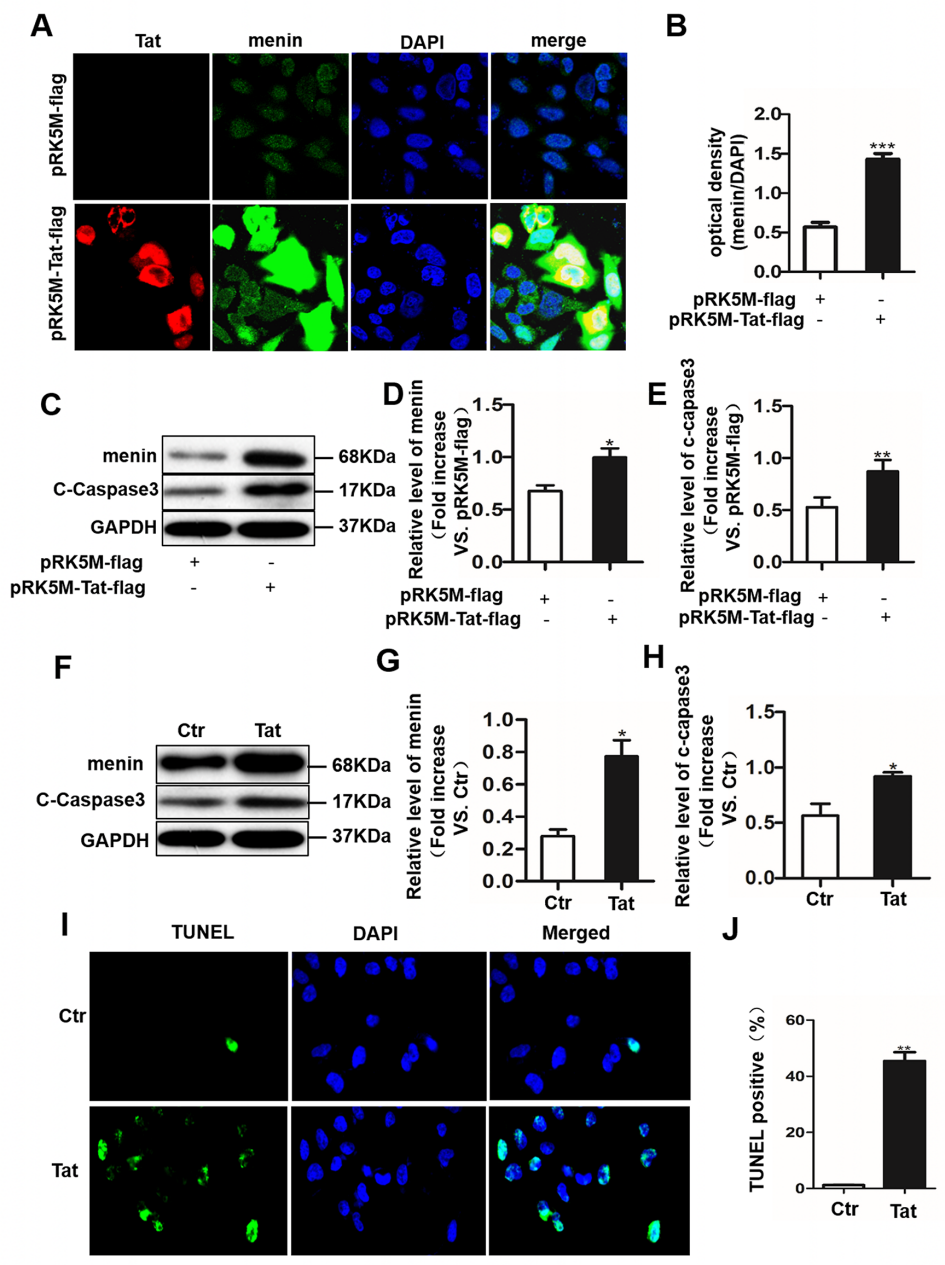
Tat induces cell apoptosis with an enhanced menin expression in SH-SY5Y cells and primary neurons SH-SY5Y cells were transfected with pRK5M-Tat-flag or pRK5M-flag for 24 hours and subjected to IHC staining. Menin (green) and Tat (red) co-localized in the nuclei of SH-SY5Y cells **A**. Optical density assay showed that menin expression is significantly increased in SH-SY5Y cells transfected with pRK5M-Tat-flag compared with the group transfected with pRK5M-flag **B**. Representative western blot were shown **C**. Optical density analysis of menin expression **D**. Optical density analysis of cleaved caspase3 expression **E**. Primary neurons were treated with or without Tat (100 ng/mL) for 48 h. Menin and cleaved-caspase 3 is significantly increased in Tat-treated neurons compared with controls **F-H**. TUNEL staining shows significantly increased apoptosis (green) in pRK5M-Tat-flag-transfected SH-SY5Y cells **I-J**. Data are plotted as mean ± SD (n = 3). *P < 0.05, **P < 0.01.

To further explore the role of menin in neuronal damage, a human neuroblastoma cell line SH-SY5Y cells were transfected with pRK5M-Tat-flag plasmid that expressing Tat protein containing 86 amino acids or pRK5M-flag vector plasmid for 24 hours, and the expression of Tat or menin were examined by immunofluorescence and western blot analysis. Immunofluorescence results showed co-localization of menin and Tat in the nuclei of SH-SY5Y cells (Figure 4A), accompanied by significantly increased menin optical density in the pRK5M-Tat-flag transfected SH-SY5Y cells when compared with control cells (Figure 4B, *P* < 0.01). Western blot results showed increased menin and cleaved-caspase 3 expression in pRK5M-Tat-flag transfected SH-SY5Y cells compared with the controls (Figure 4C-4E). These results were further confirmed in primary neurons treated with 100 ng/ml recombinant Tat protein for 24 hours, which showed that obviously increased menin and cleaved-caspase 3 expression compared with the untreated cells (Figure 4F-4H). Moreover, TUNEL assay showed that the apoptotic cells were increased in SH-SY5Y treated with Tat (100ng/ml) for 24 hours, when compared with that in the PBS treated control cells (Figure 4I-4J).

### Menin mediates tat-induced apoptosis in SH-SY5Y cells

**Figure 5:**
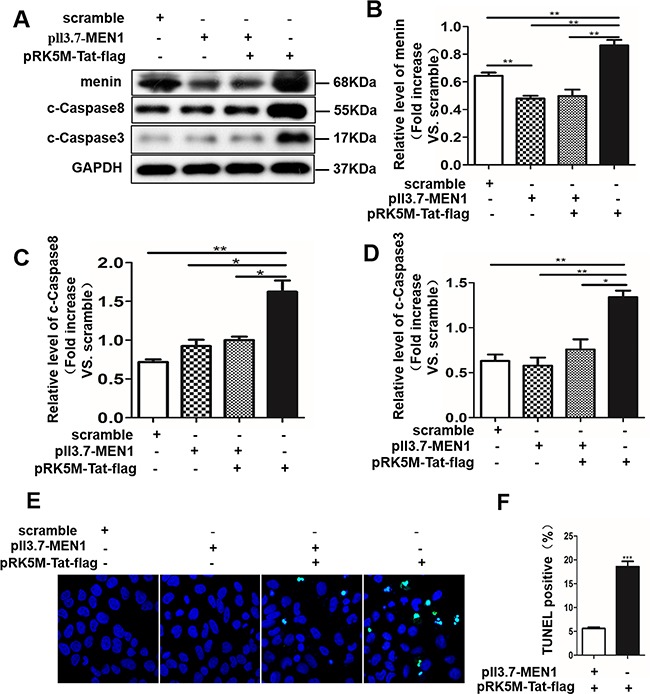
Menin mediates tat-induced apoptosis in SH-SY5Y cells MEN1 gene expression was knocked down using a specific sequence in SH-SY5Y cells stably transfected with pll3.7-MEN1-shRNA. MEN1 knockdown significantly quenches production of cleaved-caspase 3 and cleaved-caspase 8 induced by pRK5M-Tat-flag. Representative western blot **A**. and relative density of menin **B**. cleaved-caspase 8 **C**. and cleaved-caspase 3 **D**. TUNEL staining shows significantly increased apoptosis (green) in pRK5M-Tat-flag-transfected SH-SY5Y cells, and MEN1-knockdown reduced Tat-induced SY5Y cells apoptosis **E-F**. Data are plotted as mean ± SD (n = 3). *P < 0.05, **P < 0.01.

To investigate whether menin was necessary for Tat-induced neuronal apoptosis. Next, we detected cleaved-caspase 3 and cleaved-caspase 8 protein expression by transfecting SY5Y cells with a control pll3.7-scrambled-shRNA plasmid, a specific MEN1 silent plasmid pll3.7-MEN1-shRNA, pll3.7-MEN1-shRNA for 24 h followed by pRK5M-Tat-flag treatment for another 24 h, or pRK5M-Tat-flag alone for 24 h, respectively. The results showed that Tat overexpression in SY5Y cells increased cleaved-caspase 3 and cleaved-caspase 8 protein expression. Furthermore, following transfection with pRK5M-Tat-flag, the induced cleaved-caspase 3 and cleaved-caspase 8 levels were partially weakened by MEN1-knockdown (Figure 5A-5D). We used TUNEL assay to detect levels of SY5Y cells apoptosis. We observed that MEN1-knockdown reduced Tat-induced SY5Y cells apoptosis (Figure 5E-5F). These results showed that menin was necessary for Tat-induced apoptosis in SH-SY5Y cells, and aberrant overexpression of Tat may induce neuronal apoptosis through death receptor signaling pathways.

## DISCUSSION

The molecular mechanisms involved in HAND remain poorly understood. Our previous study demonstrated that injury to astrocytes, which included apoptosis, decreased EAAT-2 expression in the neuropil, and diffuse microglial activation, and limited neuronal damage, has been observed in the cortex of SIVmac239 and SHIV-RT-infected macaques. Our animals developed systemic AIDS and showed high viral loads in their plasma. There was no correlation between viral RNA load in plasma and pathological changes in cerebral cortical of SIVmac239 and SHIV-RT-infected rhesus macaques using ISH or by the polymerase chain reaction detection of SIV genomes of extracted DNA from microdissected tissue samples [10, 22–23]. In the present study, we have compared the cortical changes of two new different SIV virus, SHIV-162.P4 and SIV_sm543-3_. Our results showed that the two new virus infections had the same pathological changes in the frontal cortex with SIVmac239 and SHIV-RT infected rhesus macaques, such as goliosis of astrocytes, and neuronal apoptosis. There was no correlation between viral RNA load in plasma and menin expression in the cerebral cortex of SHIV-162.P4 and SIV_sm543-3_-infected macaques. We hypothesized that cerebral cortical changes may be related to soluble Tat.

Previous studies also demonstrated that extracellular Tat reaches the brain via the blood–brain barrier [24], crosses the membrane into adjacent cells [25], and induces neuronal apoptosis [16]. This suggests that increased levels of soluble Tat in the blood could cross the blood–brain barrier and induce astrocytic abnormalities, as well as subsequent neuronal damage. HIV-1 Tat is a potent transactivator of viral replication and a transactivator of some host genes. It is actively released from HIV-infected cells [26–27] and may act on uninfected brain-derived cells to cause a variety of effects, including activation of nuclear factor-κB [29] and neurotoxicity [29–30].

It has been recently reported that HIV-1 Tat transactivation requires menin [20], and menin is closely associated with apoptosis. Functional interactions between p53 and menin have been shown to control apoptosis in a post-irradiation INS-1 insulinoma cell line [31], and menin has been shown to up-regulate caspase 8 expression and promote TNF-alpha-induced apoptosis in murine embryonic fibroblasts [32]. Shin MH et al. reported that targeting RUNX2/Menin/MLL1/MYC axis was a feasible strategy for killing p53 defective cancer cells, and two inhibitors Mi-2 and Mi-3 of the Menin/MLL1 complex induced apoptosis in p53 defective cancer cells [33]. Nevertheless, the role of menin in HAND has not yet been reported. In the present study, we demonstrated the function of menin in SIV-infected macaques. For the first time, we showed significantly up-regulated menin expression in the frontal cortex of SHIV_-162.P4_ and SIV_sm543-3_-infected macaques in our experimental HAND model using western blot, and IHC methods. Results showed menin expression primarily in neuronal nuclei of the frontal cortex with SHIV_-162.P4_ and SIV_sm543-3_-infected macaques. Results also showed a positive correlation between the number of cleaved-caspase 3-positive cells and menin-positive cells, as well as a significant negative correlation between the number of NeuN-positive cells and menin-positive cells. So these results indicated menin was a key factor in the process of apoptosis.

Next, Tat overexpression increased menin levels and cells apoptosis in primary neurons and SY5Y cells. Conversely, menin interference decreased cleaved-caspase 3 levels in the SY5Y cells, suggesting that *in vitro* tat-induced apoptosis is menin dependent. We investigate the molecular mechanisms of menin-mediated neuronal apoptosis, and results showed that Tat overexpression increased caspase 8 protein expression in SY5Y cells. Furthermore, MEN1 knockdown partially weakened the increased cleaved-caspase 8 induced by pRK5M-Tat-flag. On the other hand, La P et al reported that menin increases caspase 8 expression by binding the caspase 8 locus in suppressing multiple endocrine neoplasia type 1 [34]. Taken together, these results suggest that Tat overexpression induces neuronal apoptosis via death receptor signaling pathways, and menin may be a regulatory factor in Tat-induced neuronal apoptosis.

In conclusion, this is the first description of increased menin expression in neurons of the frontal cortex in a macaque model of HAND, suggesting a role for menin in the development of HAND. Furthermore, results demonstrated that tat-induced apoptosis is menin dependent, and aberrant overexpression of menin induces neuronal apoptosis via death receptor signaling pathways *in vitro*, indicating that targeting menin may be potential to block the HIV-1 Tat-induced neuronal damages in HAND.

## MATERIALS AND METHODS

### SIV-infected animals

Molecularly cloned SHIV_-SF162.P4_ and SIV_sm543-3_ were used. SHIV_-162.P4_ is a T-lymphocyte-tropic virus included by the env, rev, and vpu genes from HIV-1 (SF162) (R5, MT/NSI) within the molecular clone SIV mac239. The pathogenic properties of these clones have been previously described [35–36], which includes immunosuppression and the eventual development of AIDS in rhesus macaques. SIV_sm543-3_ is a highly pathogenic heterologous virus that has also been previously described [37–38]. Thirteen rhesus macaques were screened and found to be seronegative for SIV, simian T-lymphtropic virus, B virus, and Type D retroviruses. Six macaques (*#1–6*) were intravenously inoculated with SHIV_-162.P4_ (provided by Dr. Nancy Miller, National Institute of Allergy and Infectious Diseases, National Institutes of Health, Bethesda, MD, USA), and were sacrificed at 189, 189, 163, 240, 240, and 189 weeks after inoculation, respectively. Three macaques (*#7–9*) were intravenously inoculated with SIV_sm543-3_, and were sacrificed at 77, 134, and 131 weeks after inoculation. Four uninfected macaques (*#10–13*) served as controls (Table 1). The animals were housed in individual cages and maintained according to the rules and guidelines of the Institute of Laboratory Animal Sciences of Chinese Academy of Medical Science. The animals were sacrificed at various times after infection, at a time point when they became morbid. The methods used have been previously described [10, 22–23]. This study was carried out in rigorous accordance with the recommendations in the Guide for the Care and Use of Laboratory Animals of the National Institutes of Health and in accordance with the ARRIVE (Animal Research: Reporting *In Vivo* Experiments) guidelines. The protocol was approved by the Committee on the Ethics of Animal Experiments of the Institute of Laboratory Animal Sciences of Chinese Academy of Medical Science, China.

### The viral RNA loads

According to our previously described method [10, 22–23], viral RNA loads in the peripheral blood were detected at the time of autopsy from nine SIV-infected rhesus macaques.

### Histopathological examination

Paraffin-embedded brain tissue sections (5 μm thick) were used. Immunoassays were performed using the SP kit (Maixin Biotechnology, Fuzhou, China, KIT-9720) for IHC with antibodies specific to menin (1:200, rabbit, Cell Signaling Technology, MA, USA, #6891), neuron-specific nuclear protein (NeuN) (1:200, Millipore, Darmstadt, Germany, ABN78), anti-single stranded DNA (ssDNA) (ssDNA; 1:250, Millipore, Darmstadt, Germany, MAB3299), rabbit anti-ionized calcium-binding adaptor molecule 1 (Iba1; 1:500; Wako Chemicals, Osaka, Japan), or glial fibrillary acidic protein (GFAP) (1:200, Epitomics, Burlingame, CA, USA, 2301-1), respectively, according to manual instructions.

### Double-labeled IHC and immunofluorescence

To determine the phenotype of menin-expressing cells in the SIV-infected macaques, we performed double-labeled IHC against Iba1 (Iba1; 1:500; Wako Chemicals, Osaka, Japan) or NeuN (1:200, Millipore, ABN78) using AEC (Maixin Biotechnology, Fuzhou, China, AEC-0037) or DAB/peroxidase (Maixin Biotechnology, Fuzhou, China, DAB-0031), followed by menin (1:200, rabbit, Cell Signaling Technology, MA, USA, #6891) using Vector blue/alkaline phosphatase (Maixin Biotechnology, Fuzhou, China, MAB-0141). We also performed double-labeled IHC against menin (1:200, rabbit, Cell Signaling Technology, MA, USA, #6891) using DAB/peroxidase and GFAP (1:200, Epitomics, Burlingame, CA, USA, 2301-1) with Vector blue/alkaline phosphatase using the same method.

We also performed fluorescence microscopy for double-labeled cells using antibodies specific for menin (1:200, rabbit, Cell Signaling Technology, MA, USA, #6891) and NeuN (1:100, Millipore, ABN78) in combination with Texas-red and fluorescein isothiocyanate-based detection methods for IOD analysis (Olympus IX81, Olympus Corporation, Tokyo, Japan).

We also performed immunofluorescence staining for double-label staining against menin (1:200, rabbit, Cell Signaling Technology, MA, USA, #6891) and Tat (1:200, Abcam, Cambridge, UK, ab63957) using Texas-red and fluoresce-isothiocyanate-based detection methods for IOD analysis in SY5Y cells transfected for 24 h with pRK5M-Tat-flag plasmid.

### Apoptosis assay

*In situ* terminal deoxynucleotidyl transferase-mediated dUTP-biotin end-labeling of fragmented DNA (TUNEL) and IHC of affinity-purified polyclonal rabbit immunoglobulin G directed specifically against the active form of caspase 3 (1:1000, Rabbit, Abcam, Cambridge, UK, ab13847) and the anti-ssDNA (promega, USA, G3250) were used to identify apoptotic cells according to previously reported methods [10–11].

Double-labeled IHC against ssDNA and NeuN (1:100, Millipore, ABN78) was performed to determine the phenotype of ssDNA-positive cells. We first performed ICH for ssDNA (promega, USA, G3250) using DAB/peroxidase, followed by NeuN (1:100, Millipore, ABN78) using Vector blue/alkaline phosphatase using the SP system.

To examine menin expression in cleaved-caspase 3-positive cells, we performed double-labeled IHC against menin (1:200, rabbit, Cell Signaling Technology, MA, USA, #6891) and cleaved-caspase 3 (1:400 rabbit, Abcam, Cambridge, UK, ab13847) using the same method. We first performed IHC for cleaved-caspase 3 using DAB/peroxidase, followed by menin using Vector blue/alkaline phosphatase using the SP system.

### Integrated optical density (IOD) analysis

Computer-assisted image analysis software (Image-Pro Plus, Media Cybernetics, Silver Spring, MD, USA) was used to analyze the IOD of menin-positive immunostaining detected by light microscopy (Olympus BX-URA2, Olympus Corporation, Tokyo, Japan) and double-positive immunostaining of menin/NeuN detected by fluorescence microscopy (Olympus IX81). IOD=Density(mean) * Area. The same software was also used to quantify total neurons and menin-positive neurons detected by fluorescence microscopy (Olympus IX81). We selected nine SIV-infected macaques as the infected group and compared them with four uninfected macaques (control group) for further statistical analysis of IOD. Five microscopic images (original magnification: 400×) were acquired from layers II to V of the frontal cortex from each of the thirteen selected macaques. The menin and NeuN double-labeled expression levels were scaled with a positive-stained IOD of each image using Image Pro Plus software, version 6.0 (Media Cybernetics). We also chose five images with double-NeuN/menin staining from each macaque to quantify total NeuN-positive and menin-positive neurons, as well as the mean values. We also semi-quantitatively evaluated the IHC results using the Image-Pro Plus program (Media Cybernetics). Menin-positive cells were quantified in 10 random 200× light microscopic fields of cortical layers 2–5 in the middle frontal gyrus. These findings showed an increased number of cells expressing menin, when more than 700 menin-positive cells were counted. Background levels were obtained in tissue sections immunostained in the absence of a primary antibody; corrected optical density = optical density − background. We also performed semiquantitative assessments for the following IHC findings: NeuN expression, astrocytic gliosis, caspase 3-positive cells.

### Statistical analysis

All data are expressed as mean ± SD and were analyzed using GraphPad Prism 5.0 software. Data from these studies were analyzed using the Student *t*-test to determine differences between the SIV-infected group and the control group, as well as the Spearman correlation coefficient analysis to determine the correlation between neuronal numbers and the number of menin-positive neurons in the frontal cortex of 13 macaques. *P* < 0.05 was considered statistically significant.

### Preparation and treatment of primary neurons

Pathogen-free C57BL/6 mice (female, 6~8 weeks old) were bought from the Animal Center of Xiamen Universit and were kept at there. Cortical neurons were isolated from embryonic day 15.5 (E15.5) C57BL/6 mice as previously described [24]. Briefly, the cerebral cortex was dissected, digested, and triturated to a single-cell suspension. The suspended cells were seeded and cultured in neurobasal medium containing 2% B27 supplement (Invitrogen, Carlsbad, CA, USA, 17504044). After 7 days, the neuronal cells were treated with 100 ng/mL recombinant protein Tat (ProSpec, Rehovot, Israel, hiv-105) for 24 or 48 h, followed by immunofluorescence or western blot analysis, respectively. The research was conducted strictly according to the recommendations in the Guide for the Care and Use of Laboratory Animals of the ARRIVE guidelines, and the protocol was approved by the Committee on the Ethics of Animal Experiments of the Xiamen University.

### Cell lines and treatments

SH-SY5Y cells (ATCC, Manassas, USA) were cultured in basic Dulbecco's modified Eagle's medium (DMEM) supplemented with 10% fetal bovine serum (FBS), 100 U/mL penicillin and 100 mg/mL streptomycin. Second to sixth passage cells were used in this study. pRK5M-Tat-flag was transfected into SH-SY5Y cells using Lipofectamine® 2000 Transfection Reagent (Invitrogen, Carlsbad, CA, USA, 11668019).

### Cells transfection

For the MEN1 knockdown experiment, The pll3.7-MEN1-shRNA plasmid was stably transfected into the SH-SY5Y cells for 48 h using the Lipofectamine® 2000 Transfection Reagent (Invitrogen, Carlsbad, CA, USA, 11668019) according to the manufacturer's protocol. Overexpression plasmids of pRK5M-Tat-flag and pcDNA3.1-MEN1-myc were prepared in the *E. coli* DH5α strain and extracted using Endofree Maxi Plasmid Kit (TIANGEN, Beijing, china, DP117). The SY5Y cells were respectively seeded in 24-well plates or 6-well plates, and cultured in complete DMEM supplemented with 10% FBS at 37°C in a humidified incubator in the presence of 5% CO_2_. After 24 h, the SY5Y cells were transfected with pRK5M-Tat-flag or pcDNA3.1-MEN1-myc plasmids using Lipofectamine® 2000 Transfection Reagent (Invitrogen, Carlsbad, CA, USA, 11668019). After 6 h, the cultured medium was replaced with fresh culture medium containing 10% FBS, and the cells were further incubated for another 18 h. The SY5Y cells in the 6-well plates were extracted for subsequent western blot analysis, and the SY5Y cells in the 24-well plates were selected for immunofluorescence staining.

### Western blot analysis

Total tissue protein was extracted from the fixed sample using the Qproteome FFPE Tissue Kit (Qiagen, Hilden, Germany, 37623), and SY5Y cells were extracted with RIPA lysis buffer (Boster, Wuhan, China, AR0105-100). Total protein was quantified with the BCA Protein Assay Kit (Thermo Scientific Pierce, Rockford, USA, 23227). From each sample, 20 μg protein was loaded and separated by 15% sodium dodecyl sulfate polyacrylamide gel electrophoresis, and subsequently transferred to a nitrocellulose membrane. Membrane-bound proteins were blocked with 5% milk powder, washed with phosphate-buffered saline/Tween-20 (PBST) at room temperature, and incubated with primary antibodies specific for the following: menin (1:4,000, rabbit, Abcam, Cambridge, UK, ab92443), cleaved-caspase 3 (1:1,000, rabbit, Abcam, Cambridge, UK, ab13847), cleaved-caspase 8 (1:3000, rabbit, Abcam, Cambridge, UK, ab108333), beta-actin (1:4,000, rabbit, Abcam, Cambridge, UK, ab8226), or GAPDH (1:10,000, rabbit, Abcam, Cambridge, UK, ab9485) overnight at 4°C. The blots were then washed three times with PBST, and incubated with horseradish peroxidase-labeled secondary antibodies (1:1000, rabbit, R&D, Minneapolis, MN, USA, AF1800) for 1 h at room temperature, followed by three washes in PBST. A maximum-sensitivity ECL kit (Thermo Scientific Pierce, Rockford, USA, 32106) was used to visualize the protein bands. The molecular weights of menin, cleaved-caspase 8, beta-actin, cleaved-caspase 3, and GAPDH were respectively, 68 kDa, 55 kDa, 43 kDa, 17kDa, and 37kDa.
